# Effect of Serum Cholesterol on Insulin Secretory Capacity: Shimane CoHRE Study

**DOI:** 10.1371/journal.pone.0149452

**Published:** 2016-02-16

**Authors:** Masayuki Wada, Shozo Yano, Tsuyoshi Hamano, Toru Nabika, Shunichi Kumakura

**Affiliations:** 1 Department of Internal Medicine, Okuizumo Municipal Hospital, Shimane, Japan; 2 Department of Laboratory Medicine, Shimane University Faculty of Medicine, Shimane, Japan; 3 The Center for Community-based Health Research and Education (CoHRE), Shimane, Japan; 4 Department of Functional Pathology, Shimane University Faculty of Medicine, Shimane, Japan; 5 Department of Medical Education and Research, Shimane University Faculty of Medicine, Shimane, Japan; University of Catanzaro Magna Graecia, ITALY

## Abstract

**Objectives:**

Previous studies indicate that, in addition to the blood glucose level, the lipid level in the blood may affect functions of pancreatic beta cells. In this study, we aimed to examine whether there was a relationship between the serum level of total cholesterol (TC) and the insulin secretory capacity in healthy subjects.

**Subjects and Methods:**

In participants of health examinations conducted from 2006 to 2010, we analyzed data from a total of 2,499 subjects (1,057 men and 1,442 women) after exclusion of individuals with dyslipidemia, thyroid dysfunction, diabetes, HbA1c≥6.5%, or fasting blood glucose≥126 mg/dL. Homeostasis model assessment for beta cell function (HOMA-beta) was utilized as a model representing the pancreatic beta cell function.

**Results:**

Although the serum TC level had a positive correlation with HOMA-beta in a univariate correlation analysis, after adjustment by confounding factors in a multiple regression analysis, HOMA-beta had a negative correlation with TC. This was further confirmed in a multiple logistic regression analysis, showing that higher TC was an independent risk factor for decreased insulin secretory capacity (defined as HOMA-beta≤30%) together with higher age, lower BMI, lower TG, male sex and regular alcohol intake. After the participants were stratified by BMI into three groups, the effect of TC on HOMA-beta increased along with the increase in BMI, and it was highly significant in the highest tertile.

**Conclusion:**

This cross-sectional study indicated that increased serum TC level might be related to the decrease of insulin secretory capacity in aged healthy population and that reduction of TC is more necessary in obese subjects to prevent diabetes.

## Introduction

In recent years, the number of diabetic patients was increasing explosively worldwide. In Japan, the number of people that are strongly suspected to have diabetes reached 9.5 million in 2012 [1]. Although it has been believed that insulin resistance and decreased insulin secretion are both important in the pathogenesis, diabetes is often accompanied by dyslipidemia, i.e., an elevated level of serum low density lipoprotein cholesterol (LDLC) and triglycerides (TG), and a decreased level of high density lipoprotein cholesterol (HDLC), suggesting that dyslipidemia may play an additional role in the pathogenesis of diabetes.

In fact, in experiments using *in vivo* and *in vitro* models, an elevated level of extracellular cholesterol leads to an increase in the intracellular cholesterol level [2], and this increase resulted in functional disturbance of pancreatic beta cells [3, 4]. Further, reduction of the intracellular cholesterol level by the administration of statins, an HMG-CoA reductase inhibitor, ameliorated the insulin secretory capacity in pancreatic beta cells [2]. In addition, a clinical report indicated that glucose tolerance progressively deteriorated in patients whose intracellular cholesterol level was genetically high [5]. According to these observations, it is speculated that the serum cholesterol level may affect insulin secretion and glucose metabolism through elevation of the intracellular cholesterol level of pancreatic beta cells.

In clinical settings, however, it is controversial whether statins prevent the development of diabetes; one study showed that lowering the serum cholesterol level with statins prevented the development of diabetes [6], whereas another study reported that the prevalence of diabetes was rather high in subjects with statin treatment when compared to those without the treatment [7]. To date, there are limited number of reports that examined whether serum lipid affected the insulin secretion and the glucose metabolism in humans [8–10]. Thus, the aim of this study was to obtain epidemiological evidence supporting the hypothesis that the elevation of the serum level of total cholesterol (TC) impaired the pancreatic beta cell function, namely insulin secretory capacity, in a general population.

## Materials and Methods

### Subjects

This is a cross-sectional study to examine effects of the serum level of TC on insulin secretion and insulin resistance in a general Japanese population. This study was a part of the cohort study conducted by the Center for the Community-based Health research and Education (CoHRE), Shimane University, which is performed in collaboration with counties located in rural areas of Shimane Prefecture, Japan. The Shimane CoHRE study has started at 2006, and this study was conducted using data from the baseline cohort, which is different data set from our previous reports with independent aims [11, 12].

In health examinations performed between 2006 and 2010, all individuals without severe diseases such as advanced cancer were invited to the study. Most of participants in the health examinations were over 50 years of age due to advanced aging in the area. A total of 3,306 subjects were assigned to this study. Data were collected after written informed consent was obtained from each participant.

### Ethics

Written informed consent was obtained from each participant. The study protocol was approved by the local ethics committee of Shimane University (#1555).

### Data collection

Disease histories, medication and information about lifestyle such as smoking, alcohol consumption and regular exercise were obtained by a questionnaire. Smokers were defined as subjects currently smoking daily. Similarly, habitual drinkers were defined as those drinking alcohol regularly excluding those drinking alcohol by chance. As for a habit of regular exercise, subjects self-reporting to have daily exercise were categorized as the ‘regular exercise’ group.

According to the information obtained, we excluded subjects who took medicine for dyslipidemia, diabetes and thyroid diseases, and subjects with HbA1c≥6.5% or with fasting plasma glucose (FPG) ≥126 mg/dL. Consequently, 2,499 subjects (1,057 men and 1,442 women) were included in the following analyses.

Anthropometric data were obtained in the health examinations and blood pressure was measured twice in a sitting position after 15 min of rest at the site of the examination. The lower of the two measures were taken as a representative blood pressure. Serum samples were taken after one night fasting, separated within 30 min and biochemical measurements of TC, TG, HDLC, LDLC, FPG and fasting plasma insulin (equal to fasting immunoreactive insulin, FIRI) were performed by standard methods. HbA1c was determined by HPLC. The coefficients of variation of all measurements were less than 7%.

As an indicator of insulin secretory capacity and of insulin resistance, we used the homeostasis model assessment of beta cell function (HOMA-beta) and the homeostasis model assessment for insulin resistance (HOMA-IR), respectively. HOMA-beta and HOMA-IR were calculated by the following equations; HOMA-beta=[360 × FIRI(μU / mL)]/[FPG(mg/dL)−63] and HOMA-IR =[FPG(mg/dL) × FIRI(μU/mL)]/405 [13]. HbA1c was represented according to the National glycohemoglobin standardization program value (NGSP value) [14].

### Statistics

In a multiple linear regression analysis, parameters correlated with the target variables (i.e., HOMA-beta or HOMA-IR) at r≥0.1 in a simple regression analysis were included as potential independent factors. To avoid co-linearity, one of two variables having a substantial correlation (r≥0.5) with each other was excluded from the analysis.

All data were expressed as mean±SD. All variables that did not follow a normal distribution (TG, FIRI, HOMA-beta and HOMA-IR) were analyzed after logarithmic transformation. All data were analyzed with statistical software R3.0.3 (2014.03.06), and the statistical significance was defined as *p*<0.05.

## Results

### Demographic data of the studied population

Table 1 summarizes demographic data of the studied population. HOMA-IR and HOMA-beta did not differ significantly between men and women, while several parameters relevant to diabetes (i.e., BMI, FPG and HbA1c) and to the lipid metabolism were different between the sexes. The proportion of current smokers, habitual drinkers, and subjects doing regular exercise was significantly higher in men than in women.

**Table 1:** Demographic data. BMI: body mass index, FPG: fasting plasma glucose, FIRI: fasting immunoreactive insulin, BP: blood pressure, TG: triglyceride, TC: total cholesterol, LDLC: low density lipoprotein cholesterol, HDLC: high density lipoprotein cholesterol, non-HDLC: non-high density lipoprotein cholesterol, HOMA-IR: homeostasis model assessment for insulin resistance, HOMA-beta: homeostasis model assessment for beta cell function

	All	men	women	*p* (men vs women)
**n**	2,499	1,057	1,442	
**Age (y)**	66.3±10.6	66.3±11.3	66.4±10.0	0.769
**BMI**	22.4±3.00	22.8±2.93	22.2±3.03	<0.001
**FPG (mg/dL)**	94.2±8.66	95.4±9.27	93.3±8.06	<0.001
**HbA1c (%)**	5.64±0.35	5.61±0.37	5.66±0.34	<0.001
**FIRI (μU/mL)**	3.76±4.08	3.76±4.81	3.75±3.45	0.956
**systolic BP (mmHg)**	129.5±17.2	130.1±16.7	129.1±17.6	0.172
**diastolic BP (mmHg)**	77.6±10.7	79.8±10.4	76.0±10.7	<0.001
**TG (mg/dL)**	102±57.0	108±67.1	98.1±47.9	<0.001
**TC (mg/dL)**	204±33.3	193±31.9	212±32.2	<0.001
**LDLC (mg/dL)**	122±28.7	114±27.8	128±28.0	<0.001
**HDLC (mg/dL)**	63.8±15.9	60.4±15.6	66.3±15.6	<0.001
**non-HDLC (mg/dL)**	140±32.7	133±32.4	146±31.7	<0.001
**HOMA-IR**	0.90±1.01	0.91±1.24	0.90±0.91	0.598
**HOMA-beta (%)**	43.4±44.0	42.1±54.0	44.3±34.9	0.259
**Smoking**				<0.001
**yes (n)**	265	244	21	
**no (n)**	2,234	813	1,421	
**Alcohol**				<0.001
**yes (n)**	1,101	783	318	
**no (n)**	1,398	274	1,124	
**Regular exercise**				<0.001
**yes (n)**	1,154	537	617	
**no (n)**	1,345	520	825	

### Univariate analysis between TC and glucose metabolism

In Table 2, correlations of the TC level with the parameters relating to diabetes were examined by a univariate analysis. TC had significantly positive correlations with HbA1c and BMI both in men and women (Table 2). In addition, TC was positively correlated both with HOMA-beta and HOMAR-IR not in women but in men.

**Table 2:** Correlation of TC with parameters concerning the diabetic status.

	All (n = 2,499)		Men (n = 1,057)		Women (n = 1,442)	
	r	*p*	r	*p*	r	*p*
**BMI**	0.081	<0.001	0.191	<0.001	0.097	<0.001
**HbA1c**	0.190	<0.001	0.208	<0.001	0.228	<0.001
HOMA-beta*	0.066	<0.001	0.074	<0.001	0.021	0.426
HOMA-IR*	0.091	<0.001	0.105	<0.001	0.037	0.158

*: transformed logarithmically

LDLC and non-HDLC were also positively correlated with HOMA-beta and HOMAR-IR (see Tables A and B in S3 File). TC, LDLC, non-HDLC and TG were positively correlated with FIRI and FPG (see Table C in S3 File).

### Multivariate analysis on the effect of TC on HOMA-beta, and HOMA-IR

A multiple linear regression analysis indicated that the serum TC level was inversely associated with HOMA-beta (Table 3). This association was significant even when analyzed in men or in women separately. Since the contribution of BMI on HOMA-beta was considerably high (see standardized beta in Table 3), multivariate analysis was performed after the participants were stratified by BMI into three groups with an equal number of subjects (**BMI≦21; n = 831, 21<BMI≦23.5; n = 841, and BMI>23.5; n = 827, respectively**) (Table 4). The effect of TC on HOMA-beta increased along with the increase in BMI, and, in the highest tertile (BMI>23.5), it was highly significant (beta = -0.002±0.001, p = 0.007). In contrast, the effect of TC on HOMA-IR was not significant (Table 5).

**Table 3:** Multiple linear regression analysis on HOMA-beta with various parameters. SE: standard error, std beta: standardized regression coefficient.

	All (n = 2,499)				Men (n = 1,057)				Women (n = 1,442)			
	beta	SE	std beta	*p*	beta	SE	std beta	*p*	beta	SE	std beta	*p*
**TC**	-0.001	4.62*10^−4^	-0.062	<0.001	-0.002	0.001	-0.073	0.009	-0.001	0.001	-0.054	0.023
**Age**	-0.010	0.001	-0.130	<0.001	-0.008	0.002	-0.094	0.001	-0.011	0.002	-0.151	<0.001
**BMI**	0.098	0.005	0.355	<0.001	0.105	0.009	0.341	<0.001	0.095	0.006	0.379	<0.001
**log TG**	0.337	0.035	0.184	<0.001	0.411	0.056	0.221	<0.001	0.286	0.044	0.161	<0.001
**sex**	-0.225	0.037	-0.135	<0.001								
**smoking**	0.018	0.052	0.006	0.726	-0.008	0.063	-0.004	0.902	0.110	0.147	0.017	0.454
**drinking**	-0.191	0.035	-0.114	<0.001	-0.303	0.056	-0.147	<0.001	-0.106	0.044	-0.058	0.016
**exercise**	0.061	0.030	0.037	0.045	-0.018	0.052	-0.010	0.726	0.106	0.037	0.069	0.004

**Table 4:** Multiple linear regression analysis on HOMA-beta in stratified tertile groups by BMI. SE: standard error, std beta: standardized regression coefficient.

	BMI≦21 (n = 831)				21<BMI≦23.5 (n = 841)				BMI>23.5 (n = 827)			
	beta	SE	std beta	*p*	beta	SE	std beta	*p*	beta	SE	std beta	*p*
**TC**	-0.001	0.001	-0.024	0.492	-0.002	0.001	-0.068	0.054	-0.002	0.001	-0.095	0.007
**Age**	-0.016	0.002	-0.216	<0.001	-0.011	0.003	-0.143	<0.001	-0.008	0.002	-0.117	<0.001
**log TG**	0.391	0.069	0.191	<0.001	0.380	0.060	0.211	<0.001	0.348	0.054	0.224	<0.001
**Sex**	-0.154	0.073	-0.091	0.034	-0.210	0.064	-0.135	0.001	-0.253	0.061	-0.174	<0.001
**smoking**	-0.142	0.094	-0.056	0.133	-0.025	0.093	-0.010	0.786	0.204	0.090	0.087	0.024
**drinking**	-0.189	0.066	-0.111	0.004	-0.240	0.059	-0.155	<0.001	-0.160	0.066	-0.109	0.016
**exercise**	0.035	0.056	0.021	0.528	0.082	0.051	0.053	0.109	0.036	0.062	0.024	0.564

**Table 5:** Multiple linear regression analysis on HOMA-IR with various parameters. SBP: systolic blood pressure, SE: standard error, std beta: standardized regression coefficient.

	All (n = 2,499)				Men (n = 1,057)				Women (n = 1,442)			
	beta	SE	std beta	*p*	beta	SE	std beta	*p*	beta	SE	std beta	*p*
**TC**	-0.001	0.001	-0.019	0.306	-0.001	0.001	-0.020	0.484	-0.001	0.001	-0.022	0.354
**Age**	-0.006	0.002	-0.073	<0.001	-0.006	0.002	-0.068	0.024	-0.006	0.002	-0.070	0.005
**BMI**	0.106	0.006	0.351	<0.001	0.119	0.010	0.356	<0.001	0.098	0.007	0.354	<0.001
**SBP**	0.003	0.001	0.060	0.001	0.002	0.002	0.029	0.313	0.004	0.001	0.092	<0.001
**log TG**	0.382	0.038	0.191	<0.001	0.429	0.061	0.212	<0.001	0.336	0.049	0.171	<0.001
**Sex**	-0.141	0.041	-0.078	<0.001								
**smoking**	-0.003	0.057	-0.001	0.995	-0.056	0.069	-0.024	0.412	0.160	0.164	0.023	0.327
**drinking**	-0.165	0.038	-0.091	<0.001	-0.237	0.062	-0.106	<0.001	-0.094	0.049	-0.046	0.056
**exercise**	0.084	0.033	0.046	0.012	-0.015	0.057	-0.008	0.792	0.135	0.041	0.080	<0.001

### Logistic regression analysis on impaired capacity of insulin secretion

We defined impaired capacity of insulin secretion as HOMA-beta≤ 30% according to the recent report [15], and performed a multiple logistic regression analysis to examine effects of various parameters on deficient insulin secretory activity. As shown in Table 6, TC was a significant independent factor together with other parameters such as age, male sex, BMI, TG and habitual alcohol intake. When this analysis was performed in men and in women separately, the significant effect of TC was found only in men. In women, the similar tendency was observed, which however did not reach a significant level.

**Table 6:** Multiple logistic regression analysis on impairment in insulin secretory capacity. OR: odds ratio, CI: confidence interval.

	All (n = 2,499)			Men (n = 1,057)			Women (n = 1,442)		
	OR	95%CI	*p*	OR	95%CI	*p*	OR	95%CI	*p*
**TC**	1.153	(1.047, 1.272)	0.004	1.218	(1.407, 1.420)	0.011	1.114	(0.981, 1.266)	0.097
**Age**	1.333	(1.212, 1.468)	<0.001	1.190	(1.032, 1.373)	0.017	1.457	(1.278, 1.667)	<0.001
**BMI**	0.399	(0.354, 0.448)	<0.001	0.386	(0.316, 0.468)	<0.001	0.395	(0.339, 0.459)	<0.001
**log TG**	0.709	(0.641, 0.784)	<0.001	0.672	(0.577, 0.778)	<0.001	0.722	(0.627, 0.830)	<0.001
**Sex**	1.472	(1.313, 1.650)	<0.001						
**smoking**	0.976	(0.882, 1.079)	0.628	0.972	(0.868, 1.089)	0.627	0.999	(0.736, 1.335)	0.992
**drinking**	1.254	(1.128, 1.395)	<0.001	1.481	(1.262, 1.742)	<0.001	1.116	(0.965, 1.291)	0.139
**exercise**	0.939	(0.854, 1.031)	0.188	1.408	(0.900, 1.221)	0.549	0.866	(0.766, 0.980)	0.022

## Discussion

In the present cross-sectional study, we found that the serum TC level was inversely associated with HOMA-beta independently of age, BMI and TG in a multiple linear regression analysis. This finding was further supported by the result of a multiple logistic regression analysis indicating that a high concentration of the serum TC was an independent risk factor of the impaired insulin secretion. The effect was likely to be more potent in men than in women.

The cholesterol metabolism is known to have a substantial impact on the insulin secretory capacity; in mice deficient in the gene encoding the ATP-binding cassette transporter A1 (*ABCA1*), an outward transporter of cholesterol on the cell membrane, cholesterol accumulated in pancreatic beta cells impaired the insulin secretion [2– 4]. Further, it was reported that a loss-of-function mutation in *ABCA1* reduced insulin secretory capacity without affecting insulin resistance in humans [5]. Concerning the mechanism, it was suggested that apoptotic loss of pancreatic beta cells induced by the accumulation of intracellular cholesterol resulted in reduced insulin secretory capacity [16, 17]. Since the serum cholesterol level was shown to correlate with the intracellular cholesterol level [2], an elevated serum TC level may reduce insulin secretion through elevation of the cholesterol level in the beta cells.

A couple of controversial epidemiological studies were so far reported on the association of the serum cholesterol level with functional abnormality of pancreatic beta cells as well as with abnormality in the glucose metabolism *per se* [8–10, 18, 19]; a significant correlation between LDLC and HbA1c was shown in patients with type 2 diabetes [9] and metabolic syndrome [10, 18]. By contrast, no significant correlation was found between the insulin secretory capacity and LDLC in patients with coronary artery diseases [19]. A recent study showed that TC or LDLC was inversely associated with the insulin secretory capacity in over 2,000 subjects with normal glucose tolerance [8], which was consistent with our results. In this study, however, insulin resistance seemed to be less involved in the inverse association between serum TC level and insulin secretion presumably because subjects were much younger (mean age: 43) than those in our study. We found the similar association even in aged population (mean age: 66) after adjustment with the confounding factors such as BMI and TG, suggesting that the inverse association of serum TC level with the capacity of insulin secretion would be a universal phenomenon.

Interestingly, in the present study, HOMA-beta was positively correlated with BMI and TG (Table 3). As both BMI and TG are considered to be major determinants of insulin resistance [indeed, our study indicated that these parameters showed a significant positive correlation with HOMA-IR (see Table 5)], this observation was rather unexpected. As we excluded diabetic subjects from the studied population, the participants were expected to have reserved ability of insulin secretion. Accordingly, increase in the insulin resistance due to increase in BMI or TG might be compensated by additional insulin secretion without elevation of the blood glucose level. HOMA-beta might be apparently correlated with BMI and TG through such a compensatory mechanism [20].

When all participants were divided into tertile by BMI, the correlation of TC with HOMA-beta was potentiated with increase of BMI (Table 4). This observation suggests that, to prevent diabetes, reduction of TC is more necessary in subjects with obesity [9]. Although we used TC in our analysis, the similar trend was observed when the analysis was performed with LDLC or non-HDLC instead of TC (Tables D and E in S3 File).

Although we made analysis in 2,499 healthy subjects excluded by the exclusion criteria (see Methods), such exclusion might alter statistical results. Thus, we excluded 642 subjects whose important data were lacking from 3,306 subjects to conduct the same statistics in 2,664 subjects including diabetes. Serum TC level was positively associated with HOMA-beta in all subjects (r = 0.042, p = 0.03) in a simple regression analysis, whereas an inverse association was observed in a multiple linear regression analysis (Table F in S3 File). A multiple logistic regression analysis showed that higher TC was an independent risk factor for decreased insulin secretory capacity (HOMA-beta≤30%) (Table G in S3 File), which was consistent with results after the exclusion of diabetic subjects (Table 6). These findings suggest that the results from this study may be adapted for aged general population, although medical treatment and disease status affect insulin secretion and the sensitivity.

Finally, this study has some limitations; as the effect of TC on HOMA-beta was examined under the cross-sectional study design, one cannot refer to the causal relation between TC and HOMA-beta. Although several *in vitro* studies suggested the regulatory role of the intracellular cholesterol level on the insulin secretion by pancreatic beta cells (see Introduction), it is necessary to perform a prospective study to clarify the causality. Another limitation of the study is that HOMA-beta was employed as an indicator of insulin secretory capacity. Under an epidemiological setting of the study, however, it is practically impossible to use the hyperinsulinemic-euglycemic clamp method, which is the most reliable method to estimate the insulin secretory activity. Based on the results of the present and other epidemiological studies, it may be worthy to perform a more strict clinical study using the hyperinsulinemic-euglycemic clamp.

In conclusion, in a cross-sectional study on aged healthy subjects, elevation of serum TC level was correlated with the decrease of insulin secretory capacity, and this correlation was independent of age, BMI, TG, and alcohol. Since the reduced insulin secretory capacity is a risk of the onset of diabetes in Asian [21], reduction of serum TC is expected to ameliorate a risk of diabetes. It warrants further investigation.

## Supporting Information

S1 File Data file of 2,499 subjects (XLSX)

S2 File Data file of 2,664 subjects including diabetes mellitus. (XLSX)

S3 File Supporting Tables. **Table A in S3 File. Pearson’s correlation coefficient between LDLC, BMI and glucose indices.** *: transformed logarithmically. **Table B in S3 File. Pearson’s correlation coefficient between non-HDLC, BMI and glucose indices.** *: transformed logarithmically. **Table C in S3 File. Pearson’s correlation coefficient between FIRI, FPG and lipid indices.** *: transformed logarithmically. **Table D in S3 File. Multiple linear regression analysis on HOMA-beta with various parameters. Table E in S3 File. Multiple logistic regression analysis for decreased capacity of insulin secretion. Table F in S3 File. Multiple linear regression analysis on HOMA-beta in 2,664 subjects including diabetes. Table G in S3 File. Multiple logistic regression analysis on impairment in insulin secretory capacity in 2,664 subjects including diabetes.**
(XLSX)
